# The Revolutionary Role of Ultrasound in Anaesthetic Management of Apert Syndrome: A Report of Two Cases

**DOI:** 10.7759/cureus.83148

**Published:** 2025-04-28

**Authors:** Jyoti Kanwat, Sarita Sah, Swati Das, Gopal Jalwal

**Affiliations:** 1 Anaesthesiology, All India Institute of Medical Sciences, Bathinda, Bathinda, IND

**Keywords:** airway extubation, apert syndrome, difficult airway management, high arched palate, ultrasound-guided

## Abstract

Apert syndrome, a rare congenital disorder characterized by craniosynostosis, syndactyly, and multi-system abnormalities, presents significant challenges in perioperative management. This report delineates the cases of two paediatric patients undergoing orthopaedics and hand surgeries, necessitating meticulous anaesthesia planning. Airway assessment revealed difficult intubation characteristics, necessitating the preparation of advanced airway management tools, including fibreoptic bronchoscopy and bougie-assisted intubation. Ultrasonography played a crucial role in airway evaluation, real-time vascular access, and regional anaesthesia administration. Peripheral and neuraxial blocks were used to optimize analgesia while minimizing opioid-related complications. Intraoperative care focused on maintaining normothermia, ensuring hemodynamic stability, and preventing airway-related morbidity. Extubation was carefully managed following complete neuromuscular recovery. A multidisciplinary approach, incorporating ultrasound-guided interventions, significantly enhanced the safety and efficacy of anaesthetic management. This case report emphasizes the importance of comprehensive preoperative planning and the integration of ultrasonography to mitigate complications and improve perioperative outcomes in patients with Apert syndrome.

## Introduction

Apert syndrome is a rare, congenital, autosomal dominant disorder caused by a mutation in the fibroblast growth factor receptor 2 (*FGFR2*) gene, seen in one in 65,000 births [1,2]. These patients have syndactyly of the hands and feet, craniosynostosis, midfacial anomalies, and cardiovascular and genitourinary abnormalities. Children with Apert syndrome frequently present for surgical procedures, and managing them in the operating room is often challenging. Sonoanatomy scanning of the airway before intubation, real-time ultrasound-guided peripheral nerve block, and central venous cannulation are some uses of ultrasonography that make it a beneficial tool for managing such patients [3,4].

## Case presentation

Case 1

**Figure 1:**
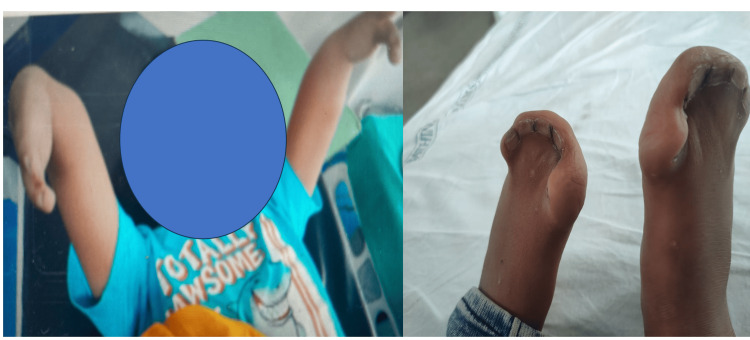
(a) Upper limb and (b) Lower limb deformity in Case 1

A seven-year-old male child weighing 20 kg who was diagnosed with Apert syndrome was scheduled for elective operative fixation of a femoral shaft fracture. The patient had an average intelligence quotient (IQ) with deformities in all four limbs (Figure 1).

In the airway examination, the patient had a high-arched palate, a short neck, and limited mouth opening with Mallampati grade III. Blood investigations, chest radiography, and electrocardiography results were within normal limits. Ultrasound scanning of the airway was performed to evaluate tracheal position, compression, and vocal cord movement. Distance from skin to epiglottis, hyomental distance ratio, and tongue base thickness were assessed to predict difficult airway, and found to be within normal range.

After confirming consent and fasting status, the patient was shifted to the operating room, where standard American Society of Anesthesiologists (ASA) monitors, along with a surface axillary temperature probe, were applied. In preparation for potential airway management challenges, a paediatric fiberoptic bronchoscope, difficult airway cart with age-appropriate endotracheal tubes, Guedel's oral airways, stylet, bougie, laryngoscope, artificial manual breathing unit (AMBU) bag, Jackson-Rees circuit, and suction apparatus were arranged. Due to the patient's limb abnormalities, intravenous cannulation presented a challenge. Under sevoflurane inhalation, while maintaining spontaneous respiration, a 4.5 French double-lumen central venous catheter was inserted into the right internal jugular vein with the help of real-time ultrasound.

**Figure 2:**
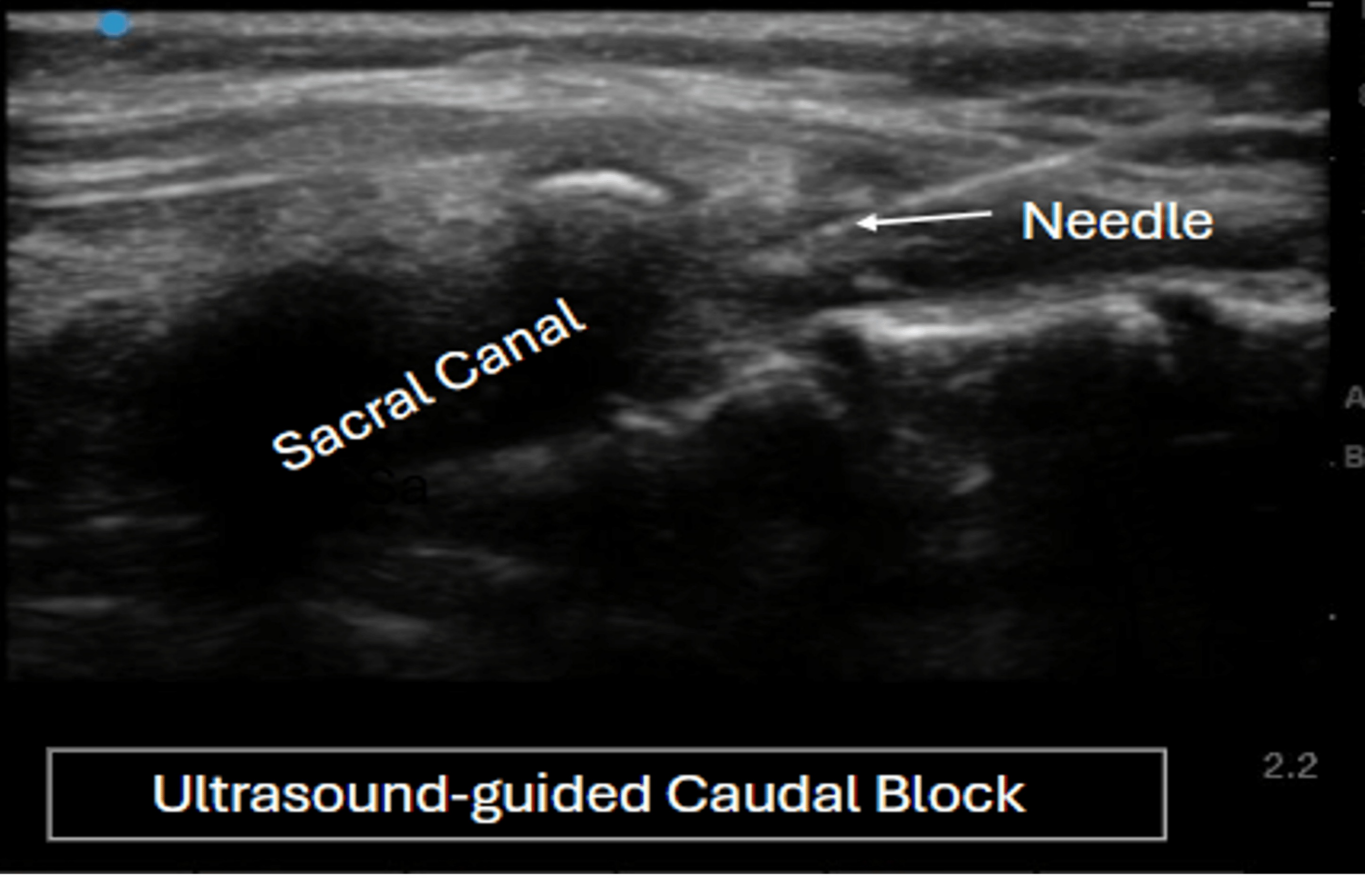
Ultrasound-guided caudal block for pain relief in Case 1

Anaesthesia was induced with the injection of propofol and fentanyl, and then cisatracurium was given after confirming adequate bag-mask ventilation. Laryngoscopy revealed a Cormack-Lehane grade III view, necessitating the use of a bougie for intubation of the trachea with a 5.5 mm cuffed endotracheal tube. For analgesia, ultrasound-guided caudal block was administered in the left lateral decubitus position, using 8 ml of 0.25% ropivacaine (Figure 2). Anaesthesia was maintained with oxygen-air and isoflurane, supplemented with intermittent doses of cisatracurium.

The surgical procedure lasted four hours, with an estimated blood loss of 300 mL, which was replaced with crystalloid solutions. Ondansetron and dexamethasone were administered prophylactically to prevent emesis and airway reactions during emergence. Neuromuscular blockade was reversed, and the patient was extubated when fully conscious and responsive to commands. Postoperatively, the patient remained pain-free and was transferred to the surgical intensive care unit for further monitoring and management.

Case 2

**Figure 3:**
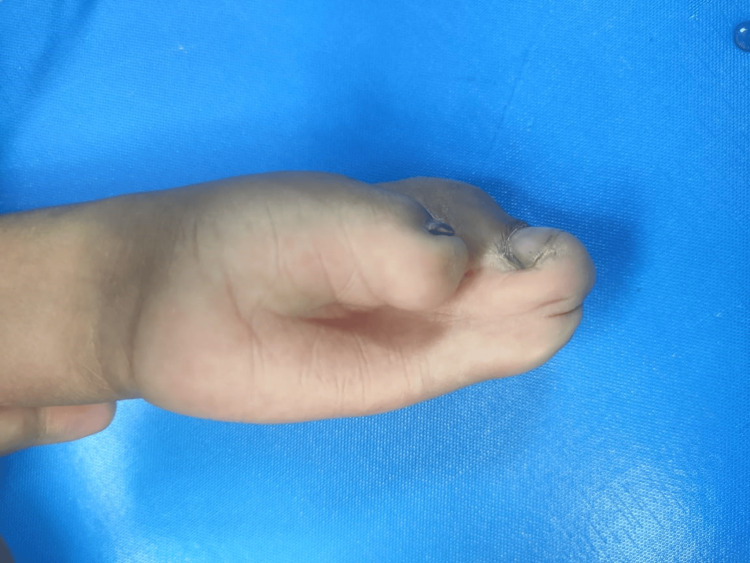
Right hand syndactyly in Case 2

An eight-year-old patient with Apert syndrome was scheduled for syndactyly release of the right hand (Figure 3). The patient had a history of seizures and a spontaneously closed atrial septal defect during the neonatal period. At the age of two years, the patient had undergone an uneventful syndactyly release. Clinical examination revealed subnormal IQ, delayed developmental milestones, macrocephaly, hypertelorism, depressed nasal bridge, left eye proptosis, down-slanting palpebral fissures, and bilateral syndactyly of hands and feet. The respiratory system examination showed pectus excavatum, but clear lung fields without added sounds or dyspnea. Airway assessment revealed a three-finger mouth opening, Mallampati grade III, and a high-arched palate.

Due to limb deformity, ultrasound-guided 22-G intravenous cannulation was performed in the cubital vein. Premedication consisted of glycopyrrolate, midazolam, and fentanyl, followed by propofol induction. Once adequate mask and ventilation were established, the muscle relaxant cisatracurium was administered. Laryngoscopy revealed a Cormack-Lehane grade II view, and a 5.5 mm cuffed endotracheal tube was used to secure the airway. An ultrasound-guided axillary nerve block was given using 10 mL of 0.2% ropivacaine for analgesia.

The surgical procedure lasted two hours with an estimated blood loss of 100 ml. Postoperative analgesia and antiemesis were managed with paracetamol and ondansetron, respectively. Prior to extubation, lignocaine and hydrocortisone were administered to mitigate airway reactivity. Upon achieving adequate tidal volume and consciousness, neuromuscular blockade was reversed, and the patient was extubated without any complications. The patient was transferred to the post-anaesthesia care unit for monitoring and discharged on the third postoperative day following an uneventful recovery period.

## Discussion

The multisystem involvement characteristic of this syndrome necessitates a comprehensive preoperative evaluation and optimization of all affected systems prior to surgical intervention. Patients with this condition typically present with reactive airways; therefore, preoperative nebulization with levosalbutamol may be beneficial in mitigating the risk of bronchospasm [5,6]. These patients are often classified as having a potentially difficult airway, warranting the use of ultrasonography to assess for airway abnormalities and the preparation of a difficult airway cart [7-9]. 

Syndactyly and limb anomalies can hinder intravenous access. Ultrasound is a valuable tool for both peripheral and central venous cannulation in such cases, potentially minimizing complications. Ultrasound-guided regional anaesthesia offers the advantage of precise peripheral and neuraxial block placement. This approach may confer additional benefits by minimizing opioid requirements and their associated side effects, thereby promoting improved respiratory function and hemodynamic stability. 

During airway management, it is advisable to maintain a deeper plane of anaesthesia for intubation. Prolonged surgical procedures may lead to hypothermia in patients due to increased blood loss and low ambient temperature of the operating room.. Maintaining normothermia is crucial and can be achieved through various interventions, including the administration of warmed intravenous fluids, application of forced-air warming devices, thermal insulation of extremities, and elevation of operating room ambient temperature [10]. Extubation should be done once the patient demonstrates full recovery from neuromuscular blockade and achieves complete consciousness to mitigate the risk of airway-related morbidity. Postoperative care necessitates a multidisciplinary approach in an intensive care unit setting to ensure optimal patient outcomes.

## Conclusions

A comprehensive understanding of Apert syndrome and meticulous anaesthetic planning are crucial before proceeding with operative interventions in affected patients. Ultrasound technology can serve as an invaluable tool in the anaesthetic management of syndromic children, facilitating successful intravenous cannulation, providing guidance for nerve blocks, and enabling detailed airway anatomy assessment. These applications of ultrasound can significantly contribute to the reduction of airway-related morbidity and mortality in this patient population.
